# Soil loss due to crop harvesting in the European Union: A first estimation of an underrated geomorphic process

**DOI:** 10.1016/j.scitotenv.2019.02.009

**Published:** 2019-05-10

**Authors:** Panos Panagos, Pasquale Borrelli, Jean Poesen

**Affiliations:** a European Commission, Joint Research Centre (JRC), Ispra, Italy; b Environmental Geosciences, University of Basel, Switzerland; c Division of Geography and Tourism, Department of Earth and Environmental Sciences, KU Leuven, Belgium

**Keywords:** Sugar beets, Potatoes, Crop harvesting, Soil loss, Erosion, Root crops

## Abstract

Over the last two decades or so, there has been many research carried out to understand the mechanics and spatial distribution of soil loss by water erosion and to a lesser extent of wind, piping and tillage erosion. The acquired knowledge helped the development of prediction tools useful to support decision-makers in both ex-ante and ex-post policy evaluation. In Europe, recent studies have modelled water, wind and tillage erosion at continental scale and shed new light on their geography. However, to acquire a comprehensive picture of soil erosion threats more processes need to be addressed and made visible to decision-makers. Since 1986, a small number of studies have pointed to an additional significant soil degradation process occurring when harvesting root and tuber crops. Field observations and measurements have shown that considerable amounts of soil can be removed from the field due to soil sticking to the harvested roots and the export of soil clods during the crop harvest. This study aims to scale up the findings of past studies, carried out at plot, regional, and national level, in order to obtain some preliminary insights into the magnitude of soil loss from cropland due to sugar beets and potatoes harvesting in Europe. We address this issue at European Union (EU) scale taking into account long-term (1975–2016) crop statistics of sugar beet and potato aggregated at regional and country levels.

During the period 2000–2016, sugar beets and potatoes covered in average ca. 4.2 million ha (3.81%) of the EU-28 arable land estimated at 110 million ha. The total Soil Loss by Crop Harvesting (SLCH) is estimated at ca. 14.7 million tons yr^−1^ in the EU-28. We estimate that ca. 65% of the total SLCH is due to harvesting of sugar beets and the rest as a result of potatoes harvesting.

## Highlights

• Soil Loss by Crop Harvesting (SLCH) is estimated at continental scale.
• SLCH is less intense compared to the water, wind, piping, tillage and gully erosion.
• The 4.2 million ha of roots crops in EU contribute to 14.7 million tons of SLCH.
• SLCH is declined by 37% between 1987–99 and 2000–2016 due to sugar beet decrease.
• SLCH may increase the overall soil loss rate in Netherlands, Belgium and Ireland.

## Graphical abstract

**Figure:**
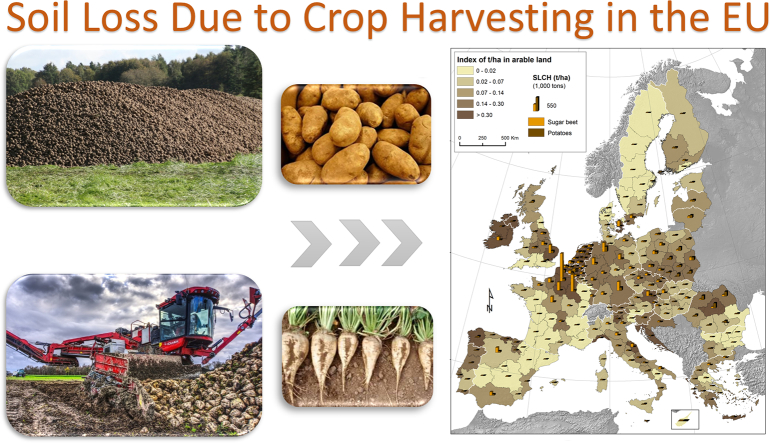

## 1 Introduction

In our days, soil erosion is recognized as one of the main processes of land degradation and a major threat to agricultural soil productivity (Montanarella, 2015; Boardman and Poesen, 2007) and thus, in many regions of the world, to societal stability. Soil erosion in croplands can further be accelerated as the result of climate change (García-Ruiz et al., 2017), land use change (Borrelli et al., 2017b) and increasing intensive agricultural management (Zhao et al., 2013).

In a recent review study, Poesen (2018) addressed the need for more research in understanding both natural and anthropogenic soil erosion processes. Most soil erosion research has focused on soil losses by water and, to a lesser extent on wind and tillage. A search in Scopus with the term “erosion and water” results in 47,428 publications, “erosion and wind” is found in ca. 8546 documents, “erosion and gully” in ca. 3463 publications while “erosion and harvest” is met only in 942 documents (Scopus, 21.9.2018).

Soil Loss due to Crop Harvesting (SLCH) is defined as the loss (or export) of top soil from arable land during harvesting of crops such as potato, sugar beet, carrot or chicory roots (Poesen et al., 2001). During the harvest of root and tuber crops, soil is sticking to the crop and is removed from the field (or it is displaced from the plot) together with stable soil clods and rock fragments (Ruysschaert et al., 2004). In addition, SLCH depends much on the soil disturbance during the harvest operation (Arnhold et al., 2014).

Several factors control the magnitude of SLCH and the rates of soil losses. The most important factors are: i) soil (soil moisture, soil texture, soil organic matter and soil structure), ii) the crop type, iii) the agronomic practices (e.g. plant density, crop yield), and iv) the harvest techniques (technology, effectiveness and velocity of harvester) (Ruysschaert et al., 2004, Ruysschaert et al., 2005). A literature review of harvest erosion (Ruysschaert et al., 2006a) collected data from different sources (e.g., FAO, literature, grey literature) and listed the mean SLCH rates per country and crop type. For sugar beets, there is quite a variation between different countries ranging from 4.7 t ha^−1^ per harvest in the United Kingdom to 10 t ha^−1^ per harvest in France (Ruysschaert et al., 2006a) while for potatoes the SCLC rates are around 3 t ha^−1^ per harvest (Ruysschaert et al., 2006b). In the literature, authors have collected data on SLCH both from experimental sites and from soil tare data provided by factories that process root and tuber crops (Ruysschaert et al., 2007b).

The United Nations (UN) Sustainable Development Goals (SDGs) explicitly identify soil resources as of crucial importance for sustainable development and food security; thus, promote the protection of soil resources to achieve the zero land degradation by 2030 (Keesstra et al., 2016). To better understand the land degradation at European scale and in specific the soil loss ones, we need to comprehensively address all erosion forms. In Europe, recent studies addressed soil losses by most soil erosion processes at continental scale and some of these quantified the soil loss rates (Table 1) but an overall estimate of SLCH is still missing. To do this, we perform a scaling up of the soil losses for the major root crops in EU.

**Table 1:** Studies on soil erosion processes (water, wind, gully, tillage, piping and landslides) at continental scale (EU).

Erosion process	Mean soil loss rates (t ha^−1^ yr^−1^)	Studies at European scale
Soil erosion by water: sheet and rill erosion	2.46 (2.7 on arable land) 1.2 (3.6 on arable land)	(Panagos et al., 2015) (Cerdan et al., 2010)
Soil erosion by water: gully erosion	Unknown	Unknown
Soil erosion by wind	0.53 (on arable land)	(Borrelli et al., 2017a)
Tillage erosion	3.3	(Van Oost et al., 2009)
Piping erosion	Unknown	Unknown
Landsliding	Qualitative assessment	(Wilde et al., 2018)

The aim of this study is to use published literature and data (Poesen et al., 2001; Ruysschaert et al., 2004, Ruysschaert et al., 2005, Ruysschaert et al., 2006b, Ruysschaert et al., 2007b) to make a preliminary estimate of the possible total soil loss by harvesting crops (SLCH) in the European Union (EU). We address the soil loss due to crop harvesting in the EU, making an attempt to complete the continental estimates of soil losses by different erosion processes. Additional objectives of this study are to: a) provide SLCH estimates at national and regional scale; b) estimate trends and c) compare soil losses due to crop harvesting with water erosion and wind erosion rates at continental scale. For this study, only two major crops were considered (i.e., a root crop: sugar beet and a tuber crop: potato) due to the availability of long time-series of crop data for these two crops, allowing us to cover the period from 1975 to 2016. Other crops such as carrot, celery and chicory have limited areas of cultivation in the EU and long-time statistical data on harvesting are not available.

Both sugar beets and potatoes are important agricultural sectors in EU agriculture. Sugar beets are cultivated to 1.8% of EU arable lands and the EU white sugar production is about 19 million tons per year (Řezbová et al., 2013). EU share to global sugar productions is about 9% and the EU is a net exporter of sugar (European Commission - EU Agricultural Outlook, 2018). Potato is the third most important food crop in terms of global consumption following wheat and rice (Devaux et al., 2014). In EU, almost 1.9 million farms grew potatoes and the total harvest was about 53 million tons for 2017 which is about 16% of the total global production (Eurostat, 2017).

## 2 Data and methods

The methodology to perform this first pan-European estimate of the total soil loss due to crop harvesting rests on crop statistics aggregated at regional and national level and soil texture data obtained through the interpolation of ca. 20,000 topsoil samples of the Land Use/Land Cover Area Frame Survey (LUCAS) (Orgiazzi et al., 2018). Other concurrent factors such as soil moisture, plant density, and harvest techniques are not considered either because relevant data are not available at EU scale or experimental data are missing.

### 2.1 Regional statistical data on crops

The European Commission Statistical Office (EUROSTAT) collects data on crop production from each country of the European Union (Eurostat, 2017). The statistics are collected annually since the 1970's and refer to all arable crops in EU (van der Velde et al., 2019). The data include areas (ha) cultivated, crop production (tons) and specific crop yield (tons ha^−1^) both at regional level and aggregated at national level.

The Nomenclature of Territorial Units for Statistics (NUTS) is the official EU database for delineating administrative units at different levels (country, region, and province). The second level in this databases (NUTS2) are geographical regions used for developing regional policies and therefore many agro-environmental indicators (greenhouse gas emissions, nutrient loads, soil erosion, soil organic carbon, among others) are presented at this scale (Yli-Viikari et al., 2007). Across the 28 EU Member States, there are 287 NUTS2 administrative units, with areas ranging from 13 km^2^ to 165,075 km^2^ and populations of 0.8–3 million inhabitants (Panagos et al., 2013). Fig. 2 presents also the delineation of the European regions (NUTS2). In the results, the old Member States refer to the 15 countries (EU-15) joining the EU before 2004 (Austria, Belgium, Denmark, Finland, France, Germany, Greece, Ireland, Italy, Luxembourg, Netherlands, Portugal, Spain, Sweden and United Kingdom). As EU-13, we refer to the Member States joining the EU after 2004 (Bulgaria, Croatia, Cyprus, Czechia, Estonia, Hungary, Latvia, Lithuania, Malta, Poland, Romania, Slovakia and Slovenia).

### 2.2 Land Use/Land Cover Area Frame Survey (LUCAS) topsoil database and derived texture maps

The Land/Use/Land Cover Area frame Survey (LUCAS) topsoil database (known as LUCAS topsoil) includes records of the physical and chemical properties of 21,682 soil samples collected in 27 EU Member States (excluding Croatia) during the period 2009–2012 (Orgiazzi et al., 2018). Among others, the physical properties include the percentages of clay, silt and sand as well as the coarse fragments (i.e. rock fragments).

Based on the point measured data, the soil texture maps were predicted using a multivariate Multivariate Adaptive Regression Splines (MARS) (Friedman, 1991). This procedure constrains the prediction of every single property as the sum of the three textural fractions should be equal to 100 percentage. The European maps of clay, silt and sand percentages are available after executing the interpolation with MARS taking also into account as spatial covariates the CORINE land cover, the Digital Elevation Model (DEM) and its derived slope, aspect and curvature (Ballabio et al., 2016).

The three soil texture maps (percentages of clay, silt and sand) can be combined in a soil texture class map using the United States Department of Agriculture (USDA) soil classification (Wösten et al., 2001). The USDA texture class map for the EU is available together with the three soil texture maps (Ballabio et al., 2016). We used the texture class map only for the EU arable lands as we applied the CORINE land cover mask to derive the EU-28 soil texture in arable lands.

### 2.3 Methodology

Our approach focuses on the two main root and tuber crops (sugar beet, potato) produced in the EU. Soil Loss by Crop Harvesting (SLCH) also takes place when harvesting other crops such as carrot (Parlak et al., 2016), garlic (Faraji et al., 2017), onions (Mwango et al., 2014), cassava (Isabirye et al., 2007), chicory (Poesen et al., 2001) and celery (Parlak et al., 2018) but the harvested area of those root crops in EU is very limited.

In a first step, we calculated the mean harvested arable land area per NUTS2 region for both sugar beets and potatoes (Eq. (1)):

(1) $$\mathrm{NUTS}2_{\mathrm{ha}}=\frac{1}{count(n)}\;\sum_{n=2000}^{2016}\mathrm{ha}$$

NUTS2_ha_ is the mean sugar beet harvested area (ha) calculated in the period 2000–2016 in order to catch the variability between regions during such a long period. The regional harvested areas of sugar beets are summed up to provide the national harvested areas (based on long-term averages). The same operation was performed for potatoes.

**Fig. 1:**
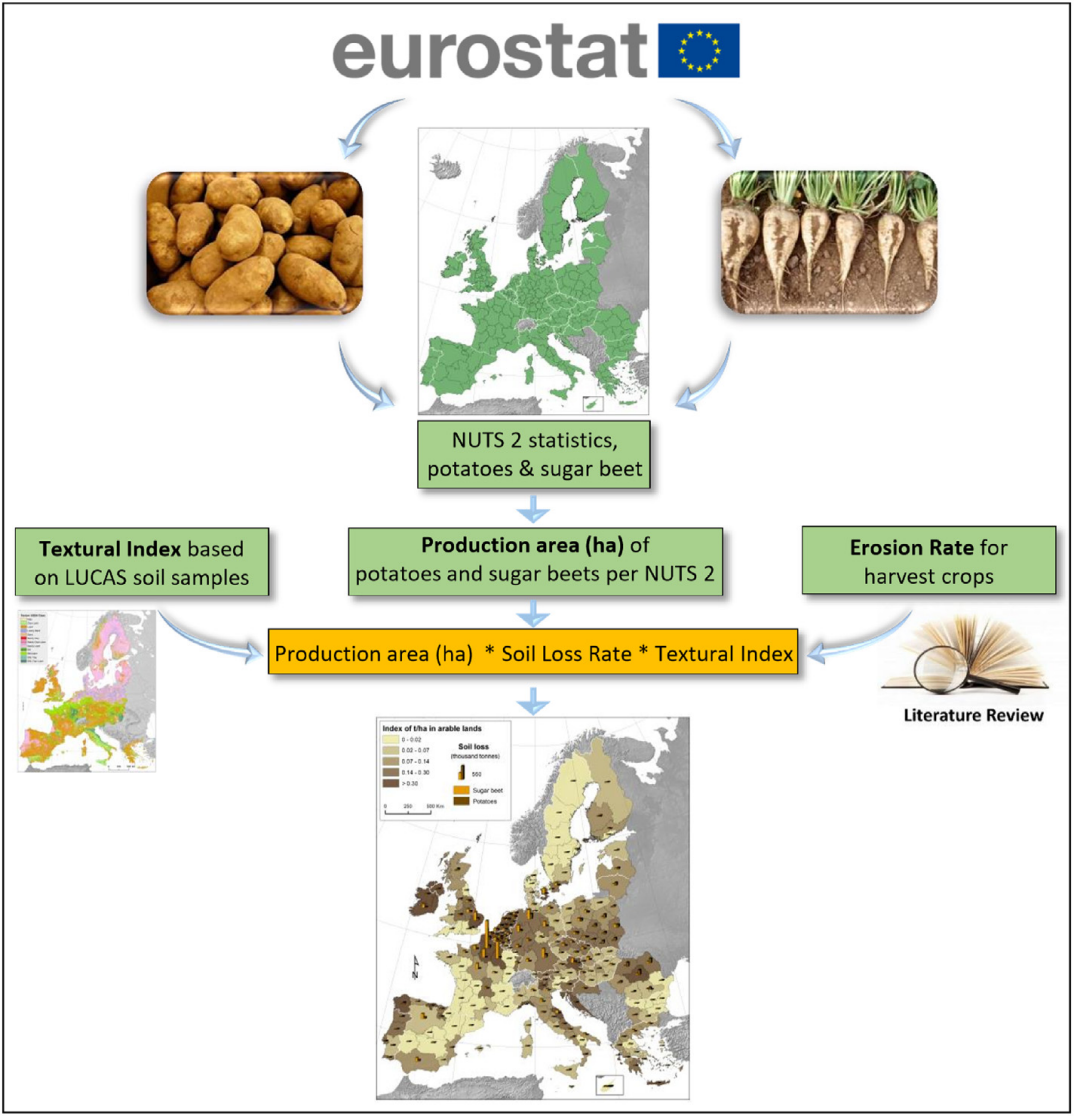
Conceptual workflow model for estimating Soil Loss due to Crop Harvesting (SLCH, tons) for potato and sugar beet at European scale.

The SLCH rates depend on several soil factors (texture, moisture and structure). In a second step, we introduced a textural index correction factor which adjusts the SLCH (Fig. 1). This textural index (dimensionless) is calculated at regional level based on the equation:

(2) $$\mathrm{Textural index}=\frac{1}{\mathrm{ha}\;\left(\mathrm{total}\right)}\sum_{k=1}^{12}{\mathrm{USDA}}_{k}*{\mathrm{ha}}_{k}$$

where k refers to each of the 12 USDA soil texture classes (Ballabio et al., 2016), USDA_k_ is a texture correction factor taking as values: 0.25 (sandy soils), 0.5 (sandy clay, sandy clay-loamy, sandy loam and silt), 0.75 (loamy sand, silt-loam, silty clay, silty clay-loam) and 1 (clay, clay-loam, loam). The value of the texture correction factor is based on available SLCH data reported in the literature (Smit et al., 1998; Poesen et al., 2001; Ruysschaert et al., 2006a) and at field plot scale in Belgium (Ruysschaert et al., 2007c). The term ha_k_ refers to the area of arable land in the specific region which has a given USDA k-value (Ballabio et al., 2016).

In a third step (Fig. 1), we applied the SLCH rates per country using available data from the literature (Ruysschaert et al., 2005, Ruysschaert et al., 2006a). Based on these data (Ruysschaert et al., 2005, Ruysschaert et al., 2006a), SLCH for the sugar beet equals 8.7 t ha^−1^ per harvest in Belgium, 6 t ha^−1^ per harvest in Germany, 10 t ha^−1^ per harvest in Denmark, 10 t ha^−1^ per harvest in France, 7 t ha^−1^ per harvest in the Netherlands, 5.6 t ha^−1^ per harvest in Spain and 4.7 t ha^−1^ per harvest in United Kingdom. In addition, we analysed data from the grey literature and found that SLCH is ca. 5 t ha^−1^ per harvest in Greece (Mikoniati, 2016) and 2.3 t ha^−1^ per harvest in Croatia (Jurišić et al., 2011). There is a tendency for smaller SLCH rates in Mediterranean countries compared to north European countries because of the dry soil conditions at harvest time which largely controls the magnitude of SLCH.

For potato, we applied a single *SLCH*_*rate*_ of 3 t ha^−1^ per harvest per harvest in all countries of the EU as proposed in the literature (Ruysschaert et al., 2006b, Ruysschaert et al., 2007a). Finally, the mean regional SLCH (t ha^−1^ yr^−1^) is estimated using the following equation:

(3) $$\text{SLCH}=\mathrm{NUTS}2_{\mathrm{ha}}*\mathrm{Textural index}*{\mathrm{SLCH}}_{\mathrm{rate}}$$

It should be underlined that both sugar beets and potatoes are generally grown on lands highly susceptible to both wind and water erosion processes (Scholz et al., 2008). In addition, both crops are generally tilled in spring and show limited canopy cover between May and July, when rain and wind erosivity are higher in Northern and Eastern Europe (Borrelli et al., 2014; Panagos et al., 2016a).

## 3 Results

The results presented here consist of a first quantitative estimation of soil losses occurring when harvesting sugar beet and potato crops in the EU.

### 3.1 Impact of texture on soil loss of root crops

The textural index (Eq. (2)) was developed to describe the impact of the soil physical properties on Soil Loss by Crop Harvesting (SLCH). According to the zonal statistics analysis performed at EU-28 arable lands (110 million ha) and the USDA soil texture map (Ballabio et al., 2016), the dominant soil texture classes (USDA classification system) are loam (33.9%), silt-loam (19%) and sandy loam (18.2%). The clay-loam represent the 12.7% of the EU arable lands, while silty clay-loam are soils are 8.7% and the loamy-sand ones the 4.4%. All the rest textural classes (sandy clay-loam, clay, silty clay, and sand) represent <1.1% each and they sum-up to 3.1% of the total arable area.

**Fig. 2:**
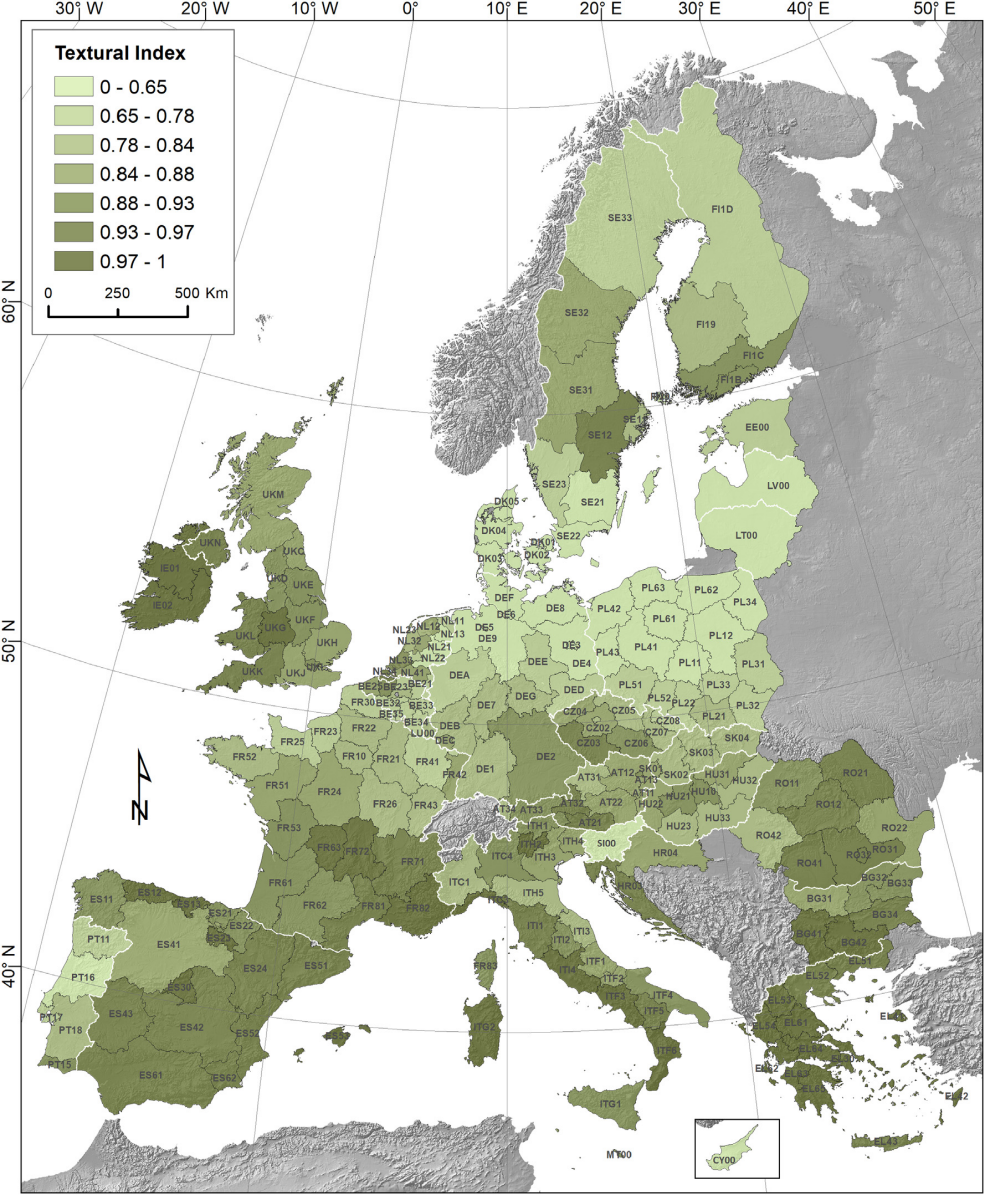
Impact of soil texture, expressed by the textural index (Eq. (2)), on Soil Loss by Crop Harvesting SLCH in the EU.

The textural index used for predicting SLCH (Eq. (2) and Fig. 2) shows an average (area-specific) value of 0.80 in the EU. It has the lowest value in Denmark (0.51), Poland (0.58), Latvia (0.64), Lithuania (0.65) and Germany (0.70), while the highest values are registered in Ireland (0.99), Greece (0.97), Spain (0.93), Romania and Bulgaria (0.92). The dominant loam soils have a textural index equal to 1 and the silt-loam soils have a corresponding index of 0.75.

### 3.2 Estimating soil losses by crop harvesting

The data on soil loss due to crop harvesting in the EU-28 are estimated based on long-term analysis for the period 2000–2016. Annually, the mean total SLCH in EU-28 is ca. 14.7 million tons; 65% of this loss is due to sugar beets and the rest 35% due to potatoes (Table 2). The European mean SLCH rate for sugar beets is 5 t ha^−1^ per harvest summing up to 9.5 million tons of soil loss for a harvested area of 1.9 million ha. The EU-28 mean SLCH rate for potatoes is 2.25 t ha^−1^ per harvest showing small variations between the EU Member States attributable to soil texture differences. With 4.2 million ha, the area under sugar beet and potato crop cover ca. 3.8% of the total arable land of the EU-28. The potatoes cover around 54% of this area while sugar beets cover the remaining 46%. However, sugar beet harvesting results in higher SLCH rates (almost double) compared to potato harvesting due to higher rates (t ha^−1^) (see above).

**Table 2:** Soil Losses due to Crop Harvesting (SLCH) in EU-28 countries (period: 2000-2016)

Country	Harvested area (1,000 ha)				Share (%) of Sugar beet & Potato in arable land area	Soil loss due to crop harvesting (1,000 tons)			Mean soil loss (ton ha^-1^ yr^-1^ ) due to sugar beet and potato harvesting calculated for the total arable land area
	Sugar beet	Potato	Sugar beet & Potato	Total Arable area		Sugar beet	Potato	Total soil loss per harvest	
Austria (AT)	44.9	22.1	67.0	1,289.5	5.19	234.1	57.8	292.0	0.23
Belgium (BE)	73.4	71.1	144.5	668.7	21.61	438.2	172.8	611.0	0.91
Bulgaria (BG)	1.4	16.6	18.0	3,857.8	0.47	6.5	48.2	54.7	0.01
Cyprus (CY)	0.0	5.2	5.2	253.4	2.06	0.0	15.6	15.6	0.06
Czechia (CZ)	62.8	41.7	104.6	2,900.6	3.60	317.6	111.8	429.4	0.15
Germany (DE)	395.3	265.9	661.2	13,560.7	4.88	1663.3	534.4	2,197.7	0.16
Denmark (DK)	38.3	28.7	66.9	2,710.2	2.47	207.2	43.6	250.8	0.09
Estonia (EE)	0.0	13.4	13.4	684.7	1.96		30.3	30.3	0.04
Greece (EL)	22.0	23.4	45.4	2,088.1	2.17	105.1	68.7	173.8	0.08
Spain (ES)	71.8	89.3	161.1	12,560.1	1.28	367.0	247.4	614.4	0.05
Finland (FI)	14.3	27.6	41.9	1,536.6	2.73	56.4	64.7	121.0	0.08
France (FR)	392.2	160.2	552.4	15,405.1	3.59	3089.0	378.4	3,467.4	0.23
Croatia (HR)	20.1	10.3	30.3	380.7	7.97	39.1	26.9	66.0	0.17
Hunary (HU)	34.1	26.8	60.9	4,805.6	1.27	172.1	67.6	239.6	0.05
Ireland (IE)	31.4	11.7	43.0	338.8	12.70	147.1	34.9	182.0	0.54
Italy (IT)	120.5	69.4	189.9	8,384.3	2.27	551.3	192.0	743.3	0.09
Lithuania (LT)	19.2	54.3	73.4	2,129.7	3.45	58.9	106.3	165.2	0.08
Luxembourg (LU)	0.0	0.6	0.6	31.5	2.00	0.0	1.5	1.5	0.05
Latvia (LV)	13.9	32.7	46.6	1,076.7	4.33	35.7	63.2	98.9	0.09
Malta (MT)	0.0	0.9	0.9	16.0	5.74		2.6	2.6	0.16
Netherlands (NL)	84.0	158.6	242.6	724.1	33.51	459.2	361.8	821.0	1.13
Poland (PL)	242.2	641.6	883.8	13,603.9	6.50	664.1	1155.2	1,819.3	0.13
Portugal (PT)	3.4	35.2	38.6	1,114.8	3.46	14.7	73.2	88.0	0.08
Romania (RO)	30.0	273.2	303.2	8,712.3	3.48	148.5	759.5	908.0	0.10
Sweden (SE)	41.3	27.7	69.1	2,944.9	2.34	111.4	55.2	166.6	0.06
Slovenia (SI)	5.6	5.2	10.8	112.9	9.56	21.5	12.8	34.3	0.30
Slovakia (SK)	24.2	16.2	40.4	1,613.7	2.50	118.5	40.0	158.5	0.10
United Kingdom (UK)	135.2	147.2	282.4	6,637.3	4.26	562.8	397.3	960.2	0.14
**EU-28**	**1,921.4**	**2,276.8**	**4,198.2**	**110,142.5**	**3.81**	**9,589.2**	**5,123.7**	**14,712.9**	**0.134**

At continental scale, it is worthy comparing the mean rate SLCH with soil loss rates of other erosion processes (water, wind). Taking into account that the mean soil loss rate from arable lands due to sheet and rill erosion is estimated at 2.67 t ha^−1^ yr^−1^ (Panagos et al., 2015), and the corresponding mean soil loss by wind erosion at ca. 0.53 t ha^−1^ yr^−1^ (Borrelli et al., 2017a), the estimated mean SLCH value of 0.13 t ha^−1^ yr^−1^ soil loss by harvesting these crops seems to play a minor role in total soil loss rates at the scale of the EU-28 compared to water and wind erosion. However, as previously observed by Borrelli et al. (2017a) in the case of wind erosion also SLCH is an unevenly distributed process that locally may assume a different relevance. According to recent studies the tolerable soil erosion, as equal to soil formation, is ca 1.4 t ha^−1^ yr^−1^ (Verheijen et al., 2009). Obviously the higher erosion rates, especially for water erosion, and in some regions for wind erosion and SLCH indicate unsustainable land use, particularly in shallow soils.

The highest areal fraction of sugar beet and potato crops at national level in the EU-28 is noticed in the Netherlands (33.5%), Belgium (21.6%) and Ireland (12.7%). This fraction is <2% in Estonia, Spain, Hungary and Bulgaria (only 0.47%). For the large EU agricultural producing countries, the share of sugar beet and potato cropping areas is relatively high in Poland (6.5%) and Germany (4.9%), medium in France and Romania (3.5%) and low in Italy (2.3%). This fraction is important as it shows the contribution of SLCH in the total soil loss by different erosional processes in arable lands of specific Member State. For example, in the Netherlands, the SLCH is relatively significant as it contributes up to 1.13 t ha^−1^ per harvest to the erosion of arable lands (similar case in Belgium with 0.91 t ha^−1^ per harvest) while in Bulgaria, Hungary, Spain and Estonia these figures are very small (<0.05 t ha^−1^ per harvest).

**Fig. 3:**
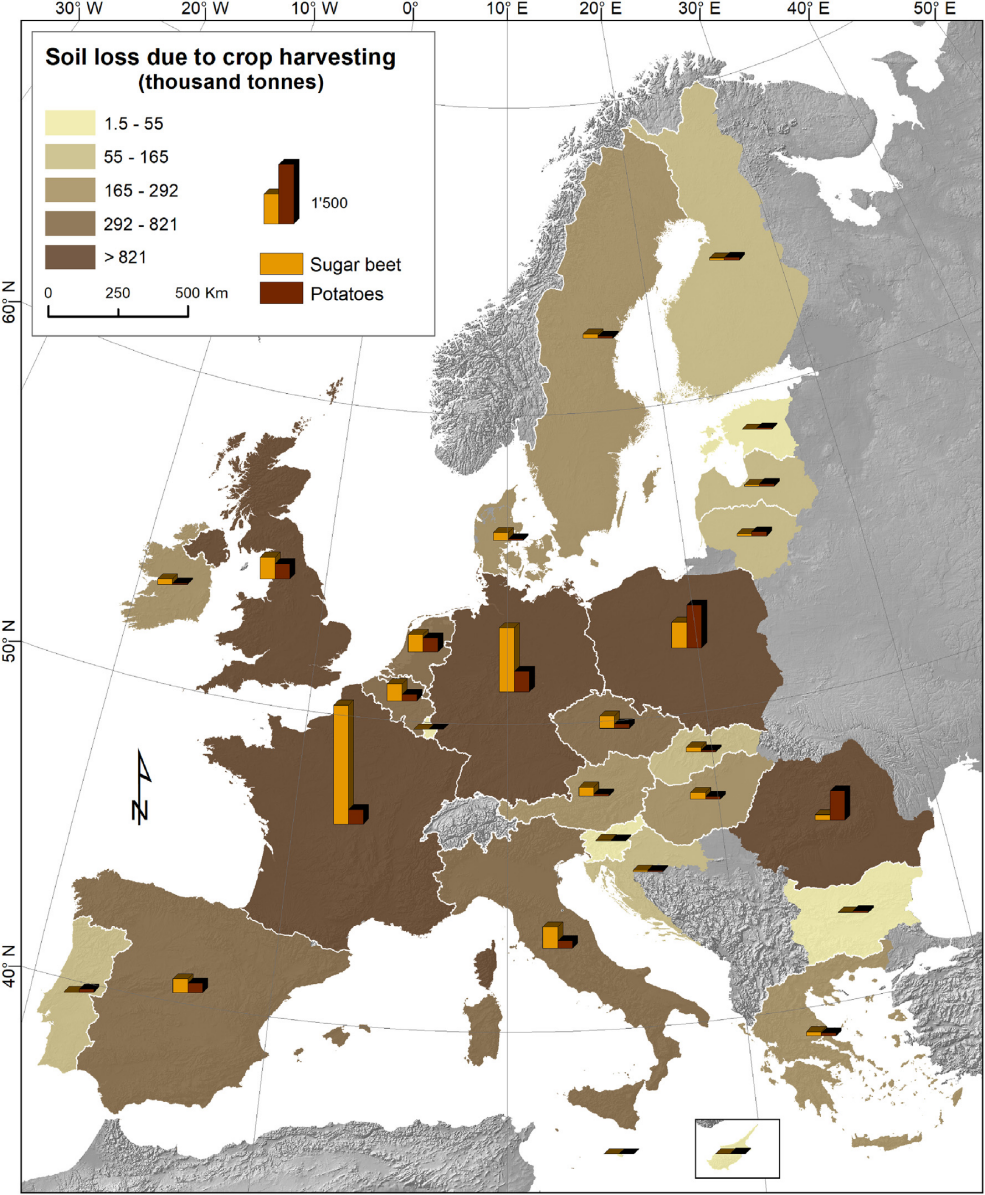
Aggregated data (at country level) on Soil Loss by Crop Harvesting (SLCH) for sugar beet and potato.

In absolute numbers, the highest SLCH in sugar beets is estimated in the largest EU agricultural producing countries. France, Germany, Poland, United Kingdom and Italy have >50% of the EU-28 arable lands and cultivate 1.28 million ha of sugar beet annually. As a result the 68% of the total SLCH in sugar beets (Table 2) is estimated for those 5 counties followed by the Netherlands, Belgium and Spain. Sugar beet is among the dominant crops in the Netherlands and Belgium where SLCH is relatively high (Fig. 3).

In most EU countries, the SLCH is higher for sugar beet compared to potato. Exceptionally, in Poland the harvested potato area is 4 times the area of harvested sugar beet and as a consequence the total SLCH is 2 times higher for potato compared to sugar beet. Also in Romania, Bulgaria, Portugal, Finland and Baltic States the potatoes have a major share in SLCH compared to sugar beets (Fig. 3). In EU-28, three countries (Poland, Romania and Germany) produce >50% of the total EU potato harvest and they contribute significantly to the SLCH due to potatoes (Fig. 3).

**Fig. 4:**
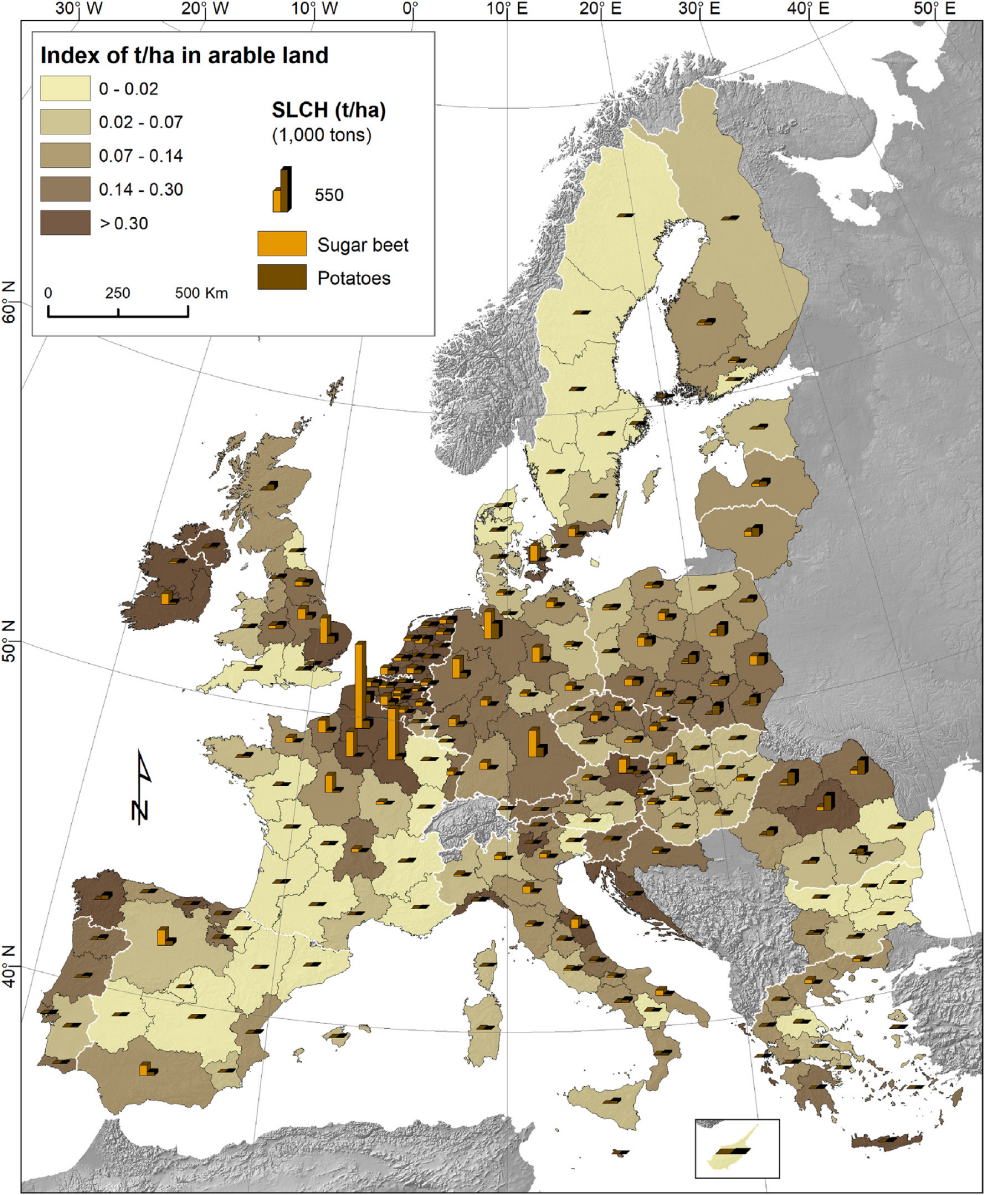
Soil Loss (1000 tons) by Crop Harvesting (SLCH) crops at regional level (NUTS2) and contribution of crop harvesting (sugar beet and potato) to area-specific soil loss (t ha^−1^) from all arable lands.

At regional level, the regions with the highest production of sugar beet and potato have the highest SLCH in absolute numbers (Fig. 4). For example the North East French regions (Picardy, Champagne-Ardenne and Nord-Pas-de-Calais) and some German regions (Saxony, Bavaria) have a total SLCH of >450,000 tons. At regional level (Fig. 4) a very high areal fraction under root and tuber crops (>45%), resulting in a large SLCH-value in regional soil loss rates, is noticed in Crete (Greece), Trento (Italy) and in the Dutch regions (Drenthe, North Brabant and Overijssel). In those regions the SLCH contributes significantly in increasing total soil loss on arable lands by at least 1 t ha^−1^. The main factors controlling SLCH at regional level are: a) the total arable land area (ha) b) the fraction of the total arable land area where root and tuber crops are grown and c) the textural index (Eq. (2); Fig. 2).

### 3.3 Trends and comparison of SLCH with past

The recent developments in the European Union Agriculture (e.g. Common Agriculture Policy, reform on EU sugar industry) and the globalisation of the markets are driving trends in cultivation of root and tuber crops. This study focuses on three periods: Recent one (2000–2016) after the adoption of the EURO currency (1999) and the finalisation of negotiations to new Member States (EU-13), 1987–1999 (period in which statistics for the new Member States are available) and the period 1975–1986 in which statistics for the old Member States (EU-15) have become available.

Comparing the last two periods, there is a significant decrease over time by 40% in SLCH for potato from 8.4 million tons to 5.1 million tons (Table 3). This negative trend in potato production is also confirmed by the historical data in the EU-15 old Member States. As a trend, a significant increase in the area of potato production (and as a result in SLCH) have been noticed only in Belgium, with a +33% during the period 2000–2016 compared to 1987–1999. Apart from Romania, all new Member States (EU-13) show a very important decrease of area under potato cultivation; in most of the Member States this decrease exceeds 50% (Table 3). Also in the Iberian countries, the share of area under potato production has been decreased >60%.

**Table 3:** Evolution of Soil Loss by Crop Harvesting (SLCH) for potatoes.

Country	1975-1986		1987-1999		2000-2016		Trend
	Harvested area (1,000 ha)	SLCH (1,000 t)	Harvested area (1,000 ha)	SLCH (1,000 t)	Harvested area (1,000 ha)	SLCH (1,000 t)	
Austria (AT)	51.7	133.8	29.3	75.8	22.1	57.8	-24%
Belgium (BE)	45.1	107.2	54.8	130.2	71.1	172.8	33%
Bulgaria (BG)			44.3	122.3	16.6	48.2	-61%
Cyprus (CY)			8.2	24.6	5.2	15.6	-37%
Czechia (CZ)			96.6	255.1	41.7	111.8	-56%
Germany (DE)	289.7	610.7	309.5	652.2	265.9	534.4	-18%
Denmark (DK)	33.2	51	39.7	61.1	28.7	43.6	-29%
Estonia (EE)			43.3	97.8	13.4	30.3	-69%
Greece (EL)	61.9	180.5	51.7	150.6	23.4	68.7	-54%
Spain (ES)	354.6	990.6	220.6	616.2	89.3	247.4	-60%
Finland (FI)	40	98.7	37.1	91.6	27.6	64.7	-29%
France (FR)	239.9	601.6	165.5	415.1	160.2	378.4	-9%
Croatia (HR)			10.3	26.2	10.3	26.2	
Hunary (HU)			52.6	132.2	26.8	67.6	-49%
Ireland (IE)	38.9	116.4	22.4	67.1	11.7	34.9	-48%
Italy (IT)	158.2	427.8	104.2	281.7	69.4	192	-32%
Lithuania (LT)			121.3	237.6	54.3	106.3	-55%
Luxembourg (LU)	1.2	2.8	0.8	2	0.6	1.5	-23%
Latvia (LV)			78.7	151.9	32.7	63.2	-58%
Malta (MT)			2.2	6.1	0.9	2.6	-58%
Netherlands (NL)	164.5	396.3	171.8	414	158.6	361.8	-13%
Poland (PL)			1,628.9	2,849.9	641.6	1,155.2	-59%
Portugal (PT)	125.5	288.3	99.9	229.4	35.2	73.2	-68%
Romania (RO)			271.3	748.3	273.2	759.5	2%
Sweden (SE)	38.5	87.8	36	82.1	27.7	55.2	-33%
Slovenia (SI)			12.7	31.2	5.2	12.8	-59%
Slovakia (SK)			45.2	111.4	16.2	40	-64%
United Kingdom (UK)	167.8	454	153.9	416.2	147.2	397.3	-5%
**TOTAL**			**3,912.7**	**8,480.2**	**2,276.8**	**5,123.7**	-40%
**EU-15**	1,810.70	4,547.3	1,497.2	3,685.6	1,138.7	2,683.8	-27%
**EU-13**			2,415.5	4,794.7	1,138.1	2,439.9	-49%

The SLCH for sugar beet has decreased significantly in the EU-28 from 15 million t yr^-1^ per harvest annually in the period 1986-1999 to 9.6 million t yr^-1^ per harvest in the period 2000-2016(-36%) (Table 4). This overall decrease was smaller (-8%)

European continent has been a major producer of potatoes with 44% of the global potato harvest in the period 1992–2010 while the EU-28 has a share of 16%. Lately the production trends turned into negative in EU-28 as the harvesting is declining (Birch et al., 2012). The reason for the reduced potato production in the EU can be related to augmented production in other countries outside the EU (i.e. China, India, Kazakhstan, Bangladesh, among others) (Hijmans, 2003) and possibly increased pathogen and pest problems due to a warming climate in Europe (Kapsa, 2008).

The SLCH for sugar beet has decreased significantly in the EU-28 from 15 million t yr^−1^ per harvest annually in the period 1986–1999 to 9.6 million t yr^−1^ per harvest in the period 2000–2016 (−36%) (Table 4). This overall decrease was smaller (−8%) comparing the data of 1975–1986 with those of 1987–1999 in EU-15. The new MS (EU-13) have the largest relative decreases in SLCH. Important to note is the decrease in SLCH for large sugar beet producing countries such as Poland (−40%), Italy (−59%), United Kingdom (−30%), Netherlands (−32%) and Belgium (−28%).

**Table 4:** Evolution of Soil Loss by Crop Harvesting (SLCH) for sugar beet.

Country	1975-1986		1987-1999		2000-2016		Trend
	Harvested area (1,000 ha)	SLCH (1,000 t)	Harvested area (1,000 ha)	SLCH (1,000 t)	Harvested area (1,000 ha)	SLCH (1,000 t)	
Austria (AT)	49.4	255.7	49	253.6	44.9	234.1	-8%
Belgium (BE)	113.6	684	101.2	609.2	73.4	438.2	-28%
Bulgaria (BG)			20.3	93.4	1.4	6.5	-93%
Cyprus (CY)							
Czechia (CZ)			105.4	556.5	62.8	317.6	-43%
Germany (DE)	411.2	1,733.4	490.8	2,068.8	395.3	1,663.3	-20%
Denmark (DK)	77.9	399.3	66.6	341.4	38.3	207.2	-39%
Estonia (EE)			0.2	0.7	0		-100%
Greece (EL)	40.7	197.5	42.5	206.5	22	105.1	-49%
Spain (ES)	221.1	1,152.7	167.6	874	71.8	367	-58%
Finland (FI)	30.9	119.4	32.8	126.8	14.3	56.4	-56%
France (FR)	549.5	4,593.8	448.4	3,748.6	392.2	3,089.0	-18%
Croatia (HR)			20.1	39.1	20.1	39.1	0%
Hunary (HU)			110.6	555.9	34.1	172	-69%
Ireland (IE)	35	164.1	33.3	156.2	31.4	147.1	-6%
Italy (IT)	273.1	1,304.6	283.6	1,355.00	120.5	551.3	-59%
Lithuania (LT)			31.7	97.2	19.2	58.9	-39%
Luxembourg (LU)							
Latvia (LV)			13.9	35.7	13.9	35.7	0%
Malta (MT)							
Netherlands (NL)	130.4	733.1	119.6	672.3	84	459.2	-32%
Poland (PL)			404.7	1,109.4	242.2	664.1	-40%
Portugal (PT)	1.3	5.6	1.6	6.9	3.4	14.7	114%
Romania (RO)			162.8	793.5	30	148.5	-81%
Sweden (SE)	52.5	187.5	53.1	189.8	41.3	111.3	-41%
Slovenia (SI)			5.8	22.3	5.6	21.5	-3%
Slovakia (SK)			43.6	214.7	24.2	118.5	-45%
United Kingdom (UK)	197.2	835.9	189.2	801.9	135.2	562.8	-30%
**TOTAL**			**2,998.5**	**14,929.8**	**1,921.4**	**9,589.2**	-36%
**EU-15**	2,183.8	12,366.6	2,079.4	11,411.2	1,468.0	8,006.7	-30%
**EU-13**			919.1	3,518.6	453.5	1,582.5	-55%

The European Union sugar reform in 2006 affected the sugar industry in many countries. The volume of sugar beet and white sugar production in EU-28 was significantly reduced. The reform affected more the sugar beet production in new EU member countries (EU-13) compared to the old EU members (EU-15) (Řezbová et al., 2013). In addition, the sugar consumption in the EU is decreasing due to consumer preferences (e.g., changing diets, decline in soft drinks consumption, increase of isoglucose consumption) (European Commission, 2018). Moreover, the developing countries such as Brazil and India gain market share due to increased production of sugar cane (Märländer et al., 2003).

**Fig. 5:**
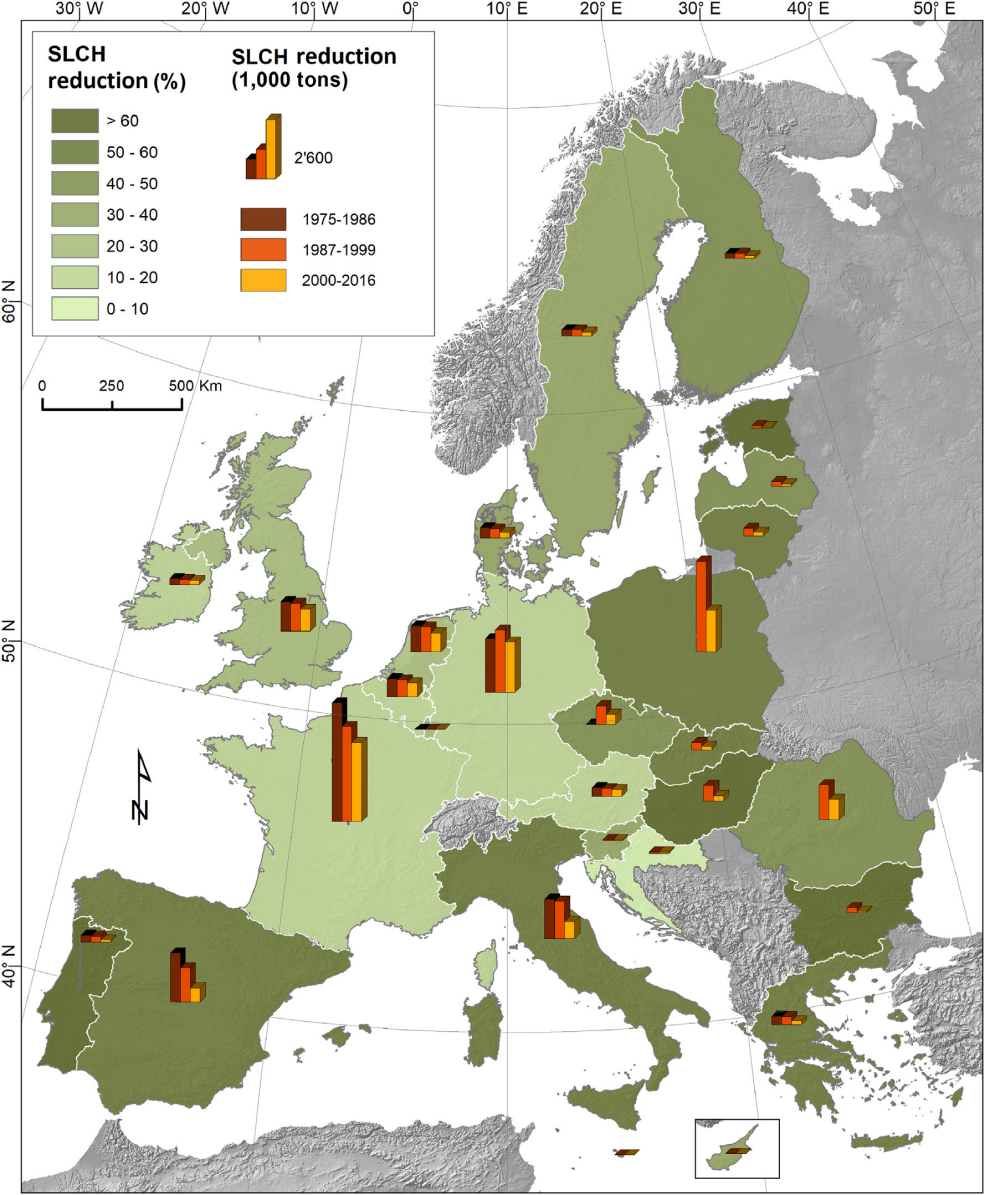
Decrease (%) of Soil Loss due to Crop Harvesting (SLCH) during the 3 study periods (1975–1986, 1987–1999 and 2000–2016).

We summed up the SLCH for sugar beet and potato and we present the aggregated data for the 3 studies periods (1975–86, 1987–99 and 2000–2016) (Fig. 5). The total SLCH in EU-28 decreased by 37.2% (23.4 million t yr^−1^ per harvest in the period 1987–99 to 14.7 million t yr^−1^ in the period 2000–16). The negative trend is confirmed in all 27 EU counties. As for Croatia we had statistical crop data only for the last period. The highest absolute decrease is found in Poland (−2.1 million t yr^−1^ per harvest) followed by Italy, Spain and France. The Mediterranean countries (Spain, Italy, Greece and Portugal) and the majority of Eastern European countries show a relative decrease exceeding 50% (Fig. 5).

## 4 Discussion

### 4.1 Soil conservation and concerns in sugar industry

According to the statistics of the EU-28 for the agricultural production in 2016 (Eurostat, 2016), the total sugar beet production is >111 million tons with an average crop yield of 74.6 t ha^−1^. As the crop harvesting also includes other materials (i.e. leafs, stones, soil and soil moisture), the mean oven-dry soil loss rate of 5 t ha^−1^ due to crop harvesting compared to the gross crop yield is in the range of the 5–10%. The mean potato yield in the EU-28 is 33.1 t ha^−1^ (Eurostat, 2016) and the mean soil loss rate of 2.25 t ha^−1^ due to crop harvesting is also in the range of 5–10%.

The rates of SLCH have large uncertainty as they depend on many factors (for a review, see Ruysschaert et al., 2004). However, a recent study estimated the mean sugar beet production in France over 88 tons ha^−1^ per harvest for the year 2016 (Heno et al., 2018). In this study, there is a reference to the mean soil losses during harvesting estimated at 5–8% (Jeanroy, 2016) which confirms our mean rate of 5 t ha^−1^ due to SLCH in sugar beets.

According to a recent article (Construction Index magazine, 2015), “*the first thing British Sugar has to do is wash all this earth off, with the result that it has more than 300,000 tons of soil to dispose of every year*”. Part of this soil is processed and sold to the market. In a similar way, The British company Topsoil states that “*annually we recycle at least 200 tons of sandy loam topsoil which is delivered with the sugar beets we buy from farmers each year*” (Topsoil, 2018).

**Fig. 6:**
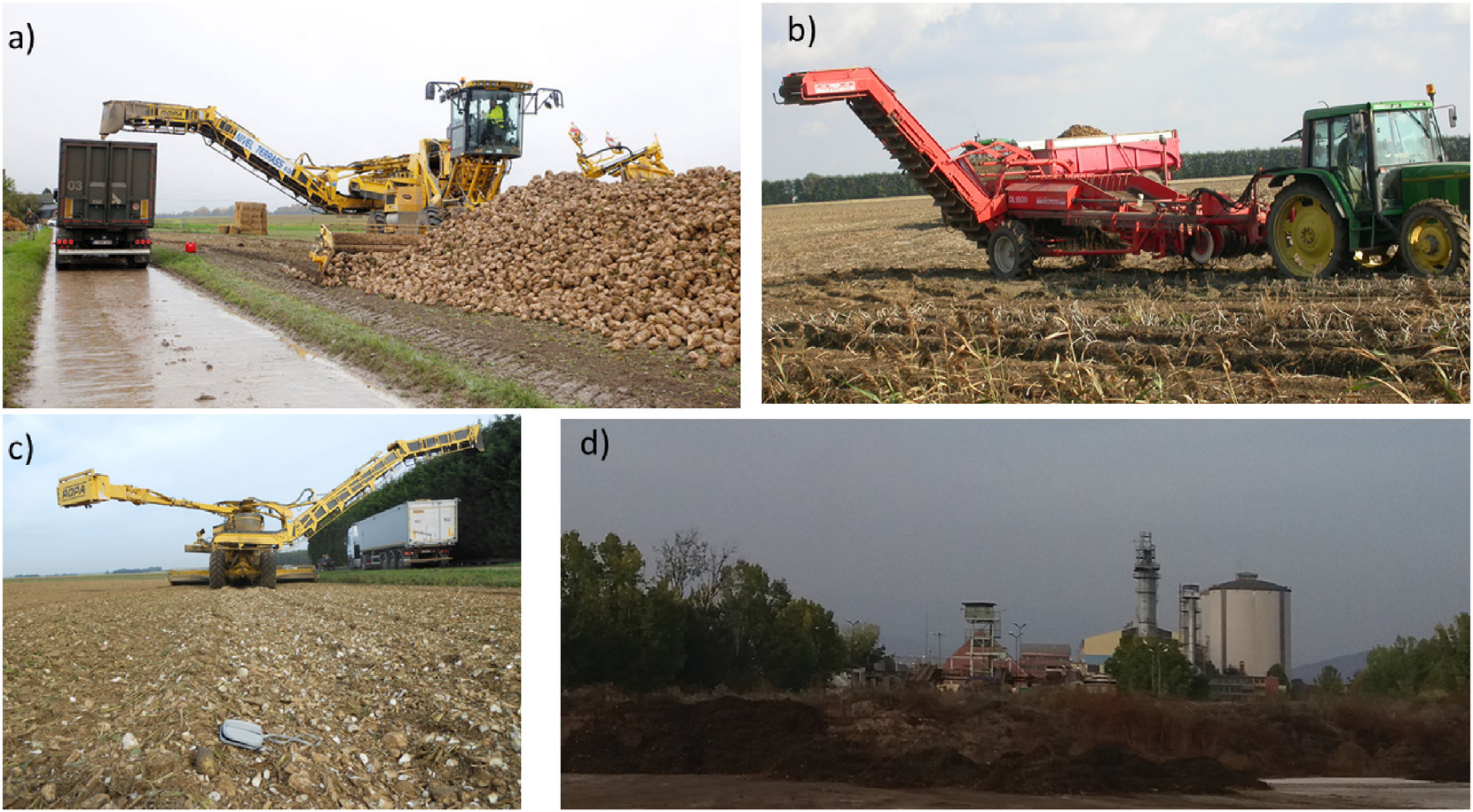
a) Mechanical cleaning of harvested sugar beets in the field to reduce soil tare, Central Belgium; b) potato harvesting, Belgium; c) topsoil and crop residues left in the field after mechanical cleaning of sugar beet, Graz-Avernas, Belgium; d) tons of soils and plant residues close to sugar Industry of Larissa, Greece.

In the sugar beet handbook by Asadi (2006), there is a concern about the main problems of sugar beet harvesting: i.e. the losses due to mixture of leaves, soil and weeds. In addition, the sugar industries have been concerned about these soil losses and related soil sustainability issues (Suedzucker, 2018). The wastewater treatment and the water consumption is a cost for the sugar industry (Vaccari et al., 2005) and they recently invest in technologies to reduce soil transferred with the tare. Therefore, industries have developed techniques and strategies to reduce SLCH: e.g. through a better cleaning of sugar beet on the cropland where the crop has been harvested, through incentives for the farmers that deliver clean sugar beet to the factories (Fig. 6).

Sugar factories and farmers widely use innovations and technological improvements for cleaning the sugar beet in the field during the last decade (Fig. 6a). As an example, the cleaning efficiency of mobile machines for beet uploading and leaning reduced the soil transported with sugar beets in the tare by at least 50% (Tuğrul et al., 2012).

### 4.2 Model and data uncertainties

Given the scale and the pioneering nature of this assessment, as well as the large temporal and spatial variability of SLCH within the EU, the results provided here do not always represent specific local conditions. In addition, the SLCH rates are long term averages and do not refer to a specific year.

The SLCH rates are much higher in mechanised (conventional) tillage systems than in reduced ones. In addition, the SLCH is proportional to the crop yields and the root-hair density (Oshunsanya et al., 2018). The rates used at national level are extracted from the literature and include uncertainties (Ruysschaert et al., 2005, Ruysschaert et al., 2006a). In addition, it was difficult to incorporate in our modelling estimates other factors that influence the SLCH rates such as soil structure, plant density or crop harvesting techniques.

Moreover, the harvesting machinery has improved during the last decade and as a result SLCH is decreasing. For example, the soil tare has decreased from 15% in 1980 to <6% in 2001 in Germany (Schulze Lammers and Strätz, 2003). Another source of uncertainty is the exclusion of other crops that generate soil losses during crop harvesting (e.g. garlic, carrots, onion, etc.) for which no statistical data on crop harvesting in the EU are currently available. However, the areas where these crops are grown are relatively small compared to the areas where sugar beet and potato are produced.

## 5 Data availability

The soil physical properties maps (Ballabio et al., 2016) are available in the European Soil Data Centre (ESDAC). The aggregated data and the regional estimates of SLCH (Fig. 2, Fig. 3, Fig. 4, Fig. 5) will be also available in ESDAC.

## 6 Summary and conclusions

Soil losses by crop harvesting have received limited attention in the soil erosion literature compared to other erosional processes. Here, we present a first estimate of Soil Loss by Crop Harvesting (SLCH) at pan-European scale to preliminary define its magnitude and spatial patterns.

Our estimates for the European Union suggest that SLCH in average removes ca. 14.7 million t yr^−1^ per harvest for the period 2000–2016. The SLCH h is relatively small compared to the losses by sheet and rill erosion estimated at 970 million t yr^−1^ (Panagos et al., 2016b) the losses due to tillage erosion at 350 million t yr^−1^ (Van Oost et al., 2009) and the losses due to wind erosion estimated at 53 million t yr^−1^ (Borrelli et al., 2017a). A comparison of the total SLCH with other soil erosion forms in the EU-28 indicate that it represents ca. 1.5%, 28%, and 4.2% of the total soil loss by water, wind and tillage erosion respectively.

The major concerns of SLCH are in countries with a dominating fraction of root and tuber crops (sugar beets and potatoes) grown in their arable land. In the Netherlands, SLCH may increase the overall soil loss rate in arable lands by 1.13 t ha^−1^ yr^−1^, in Belgium by 0.91 ha^−1^ yr^−1^ and in Ireland by 0.54 t ha^−1^ yr^−1^. In addition to those countries, SLCH contributes significantly to the total soil loss in Crete (Greece), Trento (Italy) and North East French regions. SLCH should receive more attention in regional studies where root and tuber crops are important for the local economy.

In the European Union, we estimated a significant SLCH decrease (ca. 37%) between the period 1987–99 and 2000–2016. This decrease is caused by a significant decline in the area of root and tuber crop cultivation: i.e. mainly in the new Member States (Romania, Poland, Hungary, Slovakia, Czechia, Bulgaria, Slovenia and the Baltic States). In addition to the sugar reform in 2006, the decline of sugar consumption in the EU, the increased production of root and tuber crops (sugar beet and potato) in both Asia and Latin America has contributed in reducing the harvested area of root crops in the EU from 6.9 million ha to 4.1 million ha in the last 20 years.

The technological developments in machinery harvesting, the farmers' awareness and the raising of sustainability concerns in the sugar beet industry are positive facts for further reducing the SLCH in EU. At global scale, the exponential use of biofuels in transport sector and the increased demand of root and tuber crops for energy (bioethanol) (Balat and Balat, 2009) and the increased global sugar consumption may increase the demand for sugar beets cultivation outside the European Union. This requires that SLCH should be taken into account in case of increased sugar beet production in other parts of the world.

## Conflict of interest

The authors confirm that there is no conflict of interest with networks, organisations and data centres referred to in this paper.
